# Does Early Orthodontic Treatment in Mixed Dentition Improve Long-Term Outcomes? A Systematic Review and Meta-Analysis

**DOI:** 10.3390/medicina61101854

**Published:** 2025-10-16

**Authors:** Yousef Majed Almugla, Madiraju Guna Shekhar

**Affiliations:** 1 Faculty in Orthodontics, Department of Preventive Dental Sciences, College of Dentistry, King Faisal University, Al Ahsa 31982, Saudi Arabia; yalmugla@kfu.edu.sa; 2 Faculty in Pediatric Dentistry, Department of Preventive Dental Sciences, College of Dentistry, King Faisal University, Al Ahsa 31982, Saudi Arabia

**Keywords:** early orthodontic treatment, long-term stability, relapse, mixed dentition, overjet, ANB angle, PAR score, meta-analysis, pediatric orthodontics

## Abstract

*Background and Objectives*: Early orthodontic intervention during the mixed dentition phase is commonly used to intercept developing malocclusions and potentially reduce the need for more complex treatment later. However, there is limited and conflicting evidence regarding the long-term stability of such early interventions, particularly in comparison to delayed treatment or no treatment at all. This systematic review aims to address this gap by critically evaluating and synthesizing the available evidence on the long-term skeletal and dental outcomes, following early orthodontic treatment in children aged 6–12 years versus delayed or no intervention. *Materials and Methods*: A comprehensive literature search was conducted across electronic databases (PubMed, Scopus, Web of Science, and Google Scholar) for studies published between January 1995 and April 2025. Eligible studies included randomized controlled trials, controlled clinical trials, and cohort studies involving children who underwent early orthodontic treatment using fixed or removable appliances with a minimum of one-year post-treatment follow-up. Comparator groups included no treatment or delayed treatment. Primary outcomes were long-term changes in overjet, ANB angle, and Peer Assessment Rating (PAR) scores. Meta-analyses were performed using a random-effects model, and the certainty of evidence was assessed using the GRADE framework. *Results*: A total of 18 studies were included in the systematic review, of which nine provided sufficient data on overjet for meta-analysis, with overlapping datasets available for ANB angle (*n* = 6) and PAR scores (*n* = 4). Meta-analyses showed no statistically significant long-term differences between early treatment and control groups in overjet, ANB angle, or PAR scores. Heterogeneity across outcomes ranged from low to moderate. The overall certainty of the evidence was rated as moderate, mainly due to imprecision and variability in study methods. *Conclusions*: Early orthodontic treatment provides short-term improvements in occlusal and skeletal parameters. However, current evidence does not support consistent long-term benefits over delayed intervention. Clinical decision-making should be individualized and reserved for cases with specific indications, such as functional crossbites, increased risk of dental trauma, or psychosocial concerns.

## 1. Introduction

The mixed dentition phase, typically occurring between the ages of 6 and 12 years, represents a critical period for influencing craniofacial development and guiding occlusal relationships [1]. During this transitional stage, early orthodontic intervention or interceptive treatment aims to address developing malocclusions before they progress into more complex conditions. According to the American Academy of Pediatric Dentistry (AAPD), timely recognition and management of occlusal disturbances can support the establishment of a stable, functional, and esthetic dentition [2]. Early orthodontic intervention often addresses issues like dental crowding, transverse discrepancies (e.g., crossbites), Class II malocclusions, and deleterious oral habits, aiming to guide craniofacial growth and potentially reduce the need for more extensive treatment later in adolescence [3]. However, these benefits must be critically weighed against the potential for overtreatment and the risk of relapse following growth completion. These concerns are particularly relevant when early treatment is applied broadly rather than being tailored to an individual’s specific skeletal and dental diagnosis [4]. The success of early intervention thus depends not only on the timing but also on accurate diagnosis, appropriate appliance selection, and long-term monitoring to ensure stable outcomes.

Over the past two decades, the long-term effectiveness of early orthodontic intervention has remained a subject of ongoing debate. While several studies have reported short-term benefits such as reductions in overjet, improvements in incisor alignment, and favorable skeletal changes, there is limited consensus regarding the long-term stability of these outcomes [4,5,6,7]. For instance, Fichera et al. [4] observed improved Class II skeletal relationships and incisor alignment using elastodontic appliances in growing children, with minimal relapse at one-year follow-up. Similarly, Sandhu et al. [7] documented significant improvements in overjet, overbite, and ANB angle following early treatment. However, other investigations, including those by King et al. and the two-phase clinical trial conducted by Tulloch et al., have questioned the long-term advantages of early intervention, suggesting that comprehensive treatment initiated at a later stage may yield comparable or even superior results in terms of stability and efficiency [8,9,10]. Furthermore, variations in study design, outcome measures, and follow-up durations limit meaningful comparisons across studies, making it difficult to draw definitive conclusions. These contrasting findings highlight the complexity of treatment timing decisions and underscore the need for further research that critically evaluates both the short- and long-term outcomes of early orthodontic interventions.

While individual trials and clinical guidelines often support early intervention, questions remain about its long-term stability and risk of relapse. The American Academy of Pediatric Dentistry (AAPD) emphasizes the importance of evidence-based decision-making tailored to a child’s skeletal and dental development [2]. However, the literature reveals key uncertainties: first, a lack of consensus on whether early treatment leads to stable outcomes beyond the short term; second, considerable variation in study quality, follow-up duration, and outcome definitions, which hampers comparability and generalizability [4,5,6,7,8,9,10]. Furthermore, although several systematic reviews have addressed early orthodontic treatment, most have focused on short-term outcomes such as overjet reduction or initial skeletal correction, with limited attention to post-treatment relapse, occlusal stability, or outcomes beyond one year. Additionally, prior reviews often excluded recent studies using newer treatment modalities (e.g., elastodontic appliances), and few have critically synthesized findings across both early and late intervention strategies.

This systematic review and meta-analysis seeks to bridge these gaps by evaluating the long-term stability and relapse rates of early orthodontic treatment in children aged 6 to 12 years, with a minimum follow-up of one year. Furthermore, this review compares early and late treatment or no intervention, focusing on post-treatment skeletal (e.g., ANB angle) and dental (e.g., overjet, PAR score) outcomes to provide a comprehensive synthesis that supports informed decisions on treatment timing, appliance selection, and prognosis.

## 2. Materials and Methods

This systematic review and meta-analysis was prospectively registered with the International Prospective Register of Systematic Reviews (PROSPERO; registration number: CRD420251028446, dated 9 April 2025). The review adhered to the Preferred Reporting Items for Systematic Reviews and Meta-Analyses (PRISMA) guidelines and was conducted based on the Population, Intervention, Comparator, and Outcome (PICO) framework:

- Population: Children aged 6–12 years in the mixed dentition stage undergoing early orthodontic intervention.
- Intervention: Fixed or removable orthodontic appliances used for interceptive treatment to manage developing malocclusions.
- Comparators: No treatment, placebo/attention control, or delayed treatment initiated during the permanent dentition phase.
- Outcomes: Quantitative measures of long-term treatment stability or relapse based on validated indices, including the Peer Assessment Rating (PAR) score, overjet (mm), and ANB angle (°).

### 2.1. Eligibility Criteria

Randomized controlled trials (RCTs), controlled clinical trials (CCTs), and cohort studies with a minimum of one year of post-treatment follow-up were included. The one-year threshold was set to ensure the inclusion of studies assessing long-term treatment stability and relapse, rather than short-term improvements that may not be sustained during ongoing growth and development. Studies were excluded if they involved adult populations, interventions outside the mixed dentition stage, follow-up durations of less than one year, and those which lacked extractable data (subgroup data) specific to the target age group.

### 2.2. Search Strategy

A comprehensive electronic search was conducted in PubMed (*n* = 279), Scopus (*n* = 71), Web of Science (*n* = 41), and Google Scholar (first 100 hits) for articles published between January 1995 and April 2025. Additional searches included gray literature sources such as Ovid and thesis repositories (*n* = 8). However, none of the gray literature studies met inclusion criteria due to a lack of long-term data, pediatric subgroup reporting, or appropriate control groups. Searches were restricted to English-language publications. Search strategies were customized for each database using combinations of keywords and MeSH terms related to early orthodontic treatment, stability, and relapse [Search Strategy-Supplementary File]. Reference lists of included studies were also manually screened. No authors were contacted for additional data.

### 2.3. Study Selection

One reviewer (YMA) independently screened titles, abstracts, and full texts using Rayyan systematic review software (https://www.rayyan.ai/), and duplicates were removed prior to full-text screening. To minimize potential selection bias, articles with unclear eligibility were discussed with an additional reviewer (GSM) during the full-text screening stage, and any discrepancies were resolved through discussion and consensus.

### 2.4. Data Extraction

Data were extracted independently by two reviewers (YMA and GSM) using a standardized, pre-piloted Excel form. Extracted variables included study ID, design, sample size, intervention type, comparator, outcome measures (PAR score, overjet, ANB angle), follow-up duration, and time points of assessment. While a formal inter-rater reliability statistic was not calculated, a strong agreement between reviewers was observed and all disagreements were resolved through discussion and consensus.

### 2.5. Risk of Bias Assessment

One reviewer (GSM) independently assessed the risk of bias for all included studies. The Cochrane Risk of Bias 2.0 (RoB 2) tool was used for RCTs, and the ROBINS-I tool was applied to non-randomized studies. Criteria were applied as per the guidance documents for each tool. Disagreements were resolved by consensus or, when necessary, through adjudication by a second reviewer (YMA).

### 2.6. Effect Measures

The primary effect measures were mean differences (MDs) and standardized mean differences (SMDs) with corresponding 95% confidence intervals (CIs) for continuous outcomes. All outcomes were evaluated at least one-year post-treatment.

### 2.7. Data Synthesis

Meta-analyses were performed using the DerSimonian–Laird random-effects model in Review Manager (RevMan 5.4). Statistical heterogeneity was assessed using the Higgins I^2^ statistic, with thresholds of 25%, 50%, and 75% interpreted as low, moderate, and high heterogeneity, respectively. Publication bias was not assessed, as funnel plot asymmetry is unreliable with small sample sizes; although studies included in the meta-analysis was limited, this number is considered insufficient to reliably detect small-study effects [11].

### 2.8. Certainty of Evidence

The certainty of evidence for each outcome was evaluated using the GRADE approach, considering factors such as risk of bias, inconsistency, indirectness, imprecision, and publication bias. Summary of findings tables were generated for the primary outcomes (PAR score, overjet, and ANB angle) using GRADEpro GDT software (Version 7.14.6).

## 3. Results

A total of 491 records were identified through database and registry searches: PubMed (*n* = 279), Scopus (*n* = 71), Web of Science (*n* = 41), and Google Scholar (first 100 hits). After removing 165 duplicates, 10 records were automatically excluded, and 5 were removed due to inaccessible full texts, leaving 311 records for title and abstract screening. Of the initial records, 61 were excluded as irrelevant, and 29 full-text articles were sought for retrieval. However, three reports could not be obtained in full despite multiple attempts, due to issues such as inaccessible publisher websites and unavailable archives, resulting in 26 articles available for eligibility assessment.

**Figure 1:**
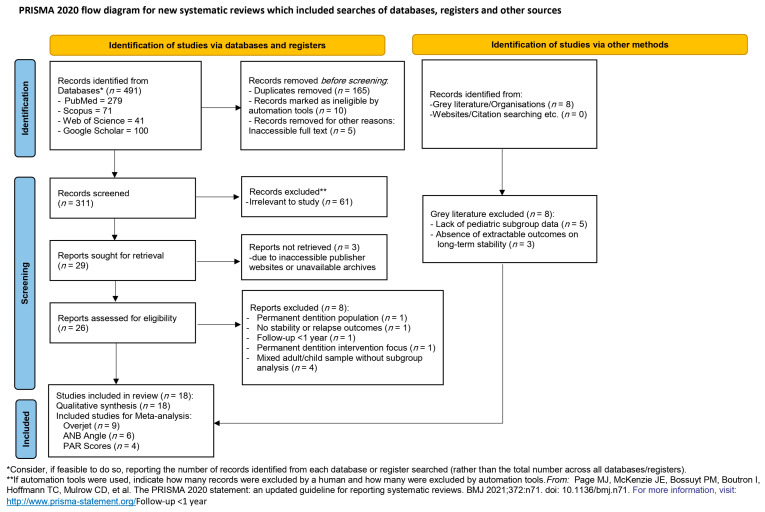
PRISMA 2020 flow diagram.

Among these 26 full-text articles, eight were excluded for the following reasons: focus on permanent dentition population (*n* = 1), lack of stability or relapse outcomes (*n* = 1), follow-up duration less than one year (*n* = 1), intervention targeting permanent dentition (*n* = 1), and mixed adult/child samples without subgroup analysis (*n* = 4). Furthermore, eight records identified through gray literature searches were excluded due to either lack of pediatric subgroup data (*n* = 5) or absence of extractable outcomes related to long-term stability (*n* = 3). Finally, 18 studies met the inclusion criteria and were included in the systematic review. Among these, nine studies provided sufficient data on overjet for meta-analysis, with overlapping datasets available for ANB angle (*n* = 6) and PAR scores (*n* = 4). The full selection process, including all reasons for exclusion, is presented in the PRISMA 2020 flow diagram (Figure 1).

### 3.1. Characteristics of Included Studies

A total of 18 studies were included in the review which comprised randomized controlled trials (*n* = 15), controlled clinical trials (*n* = 2), and one retrospective cohort study. Treatment durations ranged from 1 to 13 years, with interventions involving either removable appliances (e.g., functional appliances, facemasks, space maintainers) or fixed appliances (e.g., serial extractions, arch expansion) during the mixed dentition stage. Primary outcomes reported included overjet (mm), ANB angle (°, degrees), and Peer Assessment Rating (PAR) scores. A summary of study characteristics is presented in Tables S1 and S2 [5,6,8,10,12,13,14,15,16,17,18,19,20,21,22,23,24,25] (Supplementary Files).

### 3.2. Narrative Analysis of Included Studies

The 18 included studies demonstrated considerable clinical and methodological heterogeneity in terms of sample sizes, appliance types, treatment protocols, and follow-up durations. Interventions ranged from fixed appliances (e.g., bonded expanders, serial extractions) to removable functional appliances (e.g., Frankel II, Bionator, Twin Block). Studies with follow-up periods exceeding two years yielded more reliable insights into relapse patterns and skeletal changes (or long-term stability).

A general trend was observed wherein early orthodontic treatment led to temporary improvements in outcomes such as overjet and ANB angle. However, these benefits frequently diminished or relapsed over time, particularly in the absence of adequate retention or in cases of unfavorable growth patterns. Studies conducted by Tulloch et al. and Baccetti et al. found no clinically significant long-term skeletal advantage of early functional appliance use compared to treatment initiated at later stages [5,18]. Regarding dental arch stability, King et al. and Keski-Nisula et al. reported moderate long-term retention of overjet correction [8,14], whereas Anne Mandall et al. observed significant relapse during adolescence [17]. Another study by Quinzi et al. (2023), evaluated serial extraction as an interceptive approach, and reported mixed outcomes in post-treatment tooth alignment [12].

The variability in appliance types, outcome measurement tools (e.g., PAR index vs. cephalometric measures), and study/methodological quality limited direct comparison, highlighting the need for outcome-specific meta-analyses. Overall, the evidence suggests short-term benefits of early orthodontic treatment, but long-term stability, especially in skeletal dimensions, remains uncertain without appropriate retention strategies.

### 3.3. Risk of Bias

Risk of bias in randomized controlled trials was assessed using the Cochrane RoB 2.0 tool. Among the 15 RCTs evaluated, most studies were rated as having low risk of bias in key domains such as the randomization process, deviations from intended interventions, and completeness of outcome data. However, a substantial number of studies were rated to have “some concerns” in the domains of outcome measurement and selective reporting, primarily due to limited blinding of outcome assessors or lack of pre-registered protocols.

**Figure 2:**
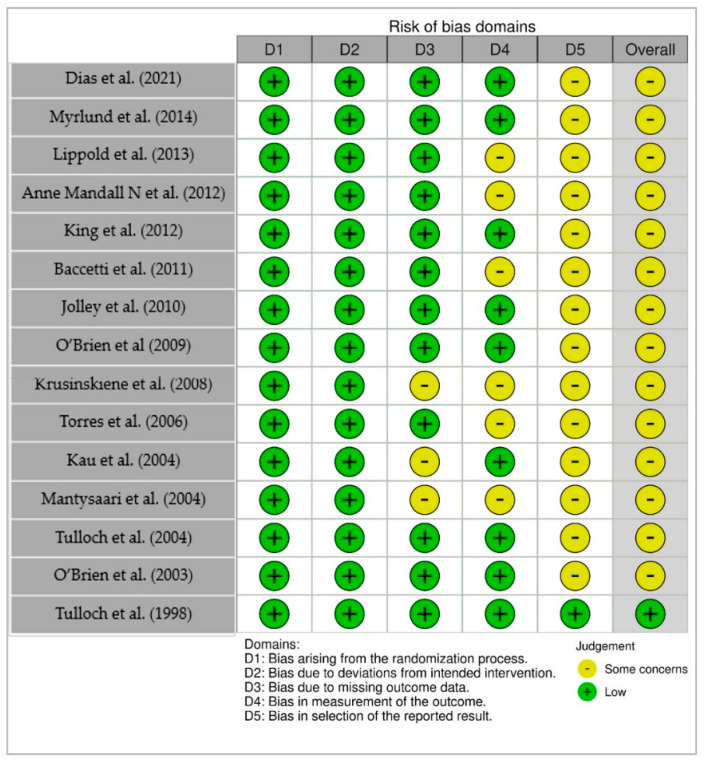
Risk of Bias Assessment for Randomized Controlled Trials (RoB 2.0). Traffic light plot summarizing judgments across five domains for included RCTs [5,6,8,10,13,15,16,17,18,19,21,22,23,24,25].

Notably, only one study (Tulloch et al., 1998) [10] was rated as low risk of bias across all five domains, reflecting its methodological rigor. In contrast, other studies, such as Krusinsksene et al. (2008) [21] and Mantysaari et al. (2004) [24], raised concerns related to subjective outcome assessment (e.g., reliance on clinical judgment without blinding) and incomplete reporting of planned analyses. A visual summary of the domain-level assessments using a traffic light plot is presented in Figure 2. While the overall quality of included RCTs is moderate to high, the presence of some methodological concerns in multiple studies suggests that the pooled results should be interpreted with caution, especially for outcomes where assessment blinding or selective reporting may have introduced bias.

**Figure 3:**
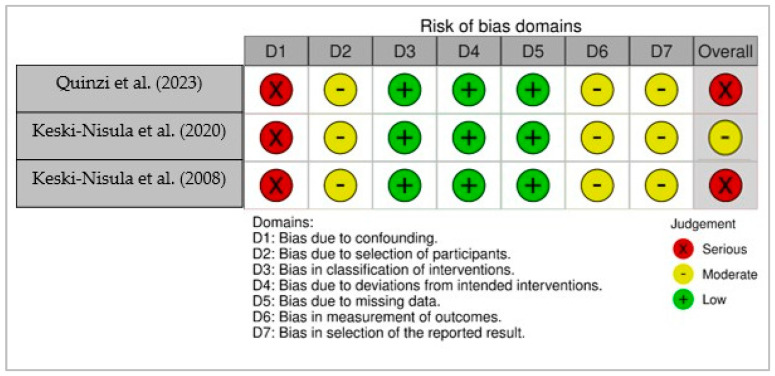
Risk of bias assessment for non-randomized studies (ROBINS-I) shown as a traffic light plot across seven domains [12,14,20].

Non-randomized studies were evaluated with the ROBINS-I tool. Three studies (Quinzi et al., 2023; Keski-Nisula et al., 2020; Keski-Nisula et al., 2008) [12,14,20] were included in this evaluation. All studies demonstrated a high risk of bias due to confounding and moderate risk in domains related to participant selection, outcome measurement, and selective reporting. Two of the studies were judged to have an overall high risk of bias, and one study was rated as having a moderate overall risk. A visual summary of the risk of bias assessment for non-randomized studies is presented in Figure 3, which illustrates bias judgments across seven domains.

### 3.4. Meta-Analysis Results

#### 3.4.1. Overjet (mm)

**Figure 4:**
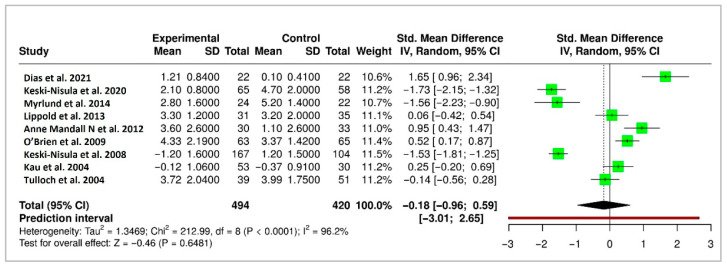
Forest plot of meta-analysis for overjet reduction following interceptive orthodontic treatment. Green blocks indicate study effect sizes and weights. Horizontal lines are the 95% confidence intervals. The red vertical line is the no-effect line (0). The black rhombus represents the pooled effect estimate with its confidence interval [5,6,13,14,15,16,17,20,23].

A meta-analysis of 9 randomized controlled trials (RCTs) evaluating long-term changes in overjet yielded a pooled standardized mean difference (SMD) of −0.18 mm (95% CI: −0.96 to 0.59), indicating no statistically significant difference in long-term stability between early treatment and control groups. However, the analysis showed substantial heterogeneity (Higgins’ I^2^ = 96.2%), reflecting variability among studies. The forest plot for overjet is shown in Figure 4.

#### 3.4.2. ANB Angle (°)

**Figure 5:**
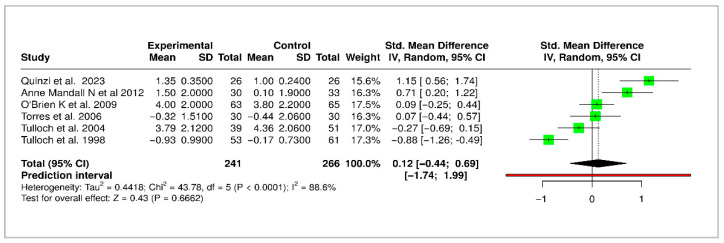
Forest plot of meta-analysis for ANB angle correction following interceptive orthodontic treatment. Green blocks indicate study effect sizes and weights. Horizontal lines are the 95% confidence intervals. The red vertical line is the no-effect line (0). The black rhombus represents the pooled effect estimate with its confidence interval [5,6,10,12,17,22].

A meta-analysis of 6 RCTs evaluated the long-term changes in ANB angle following early orthodontic treatment. The pooled SMD was −0.12 (95% CI: −0.44 to 0.69), indicating no statistically significant differences in skeletal changes between the early intervention and control groups. Substantial heterogeneity was observed (Higgins’ I^2^ = 88.6%), suggesting inconsistency across studies. The forest plot for ANB angle is shown in Figure 5.

#### 3.4.3. Peer Assessment Rating Score

**Figure 6:**
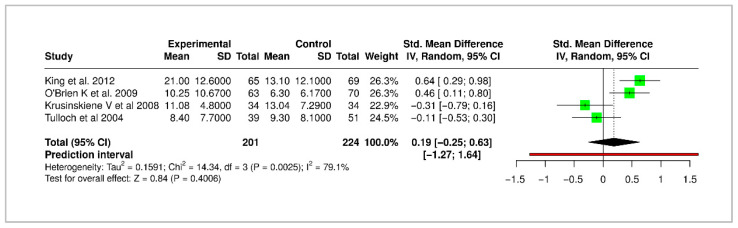
Forest plot of meta-analysis for PAR score improvement in treated vs. untreated groups. Green blocks indicate study effect sizes and weights. Horizontal lines are the 95% confidence intervals. The red vertical line is the no-effect line (0). The black rhombus represents the pooled effect estimate with its confidence interval [5,6,8,21].

Four studies reported long-term changes in Peer Assessment Rating (PAR) score outcomes, with a pooled SMD of 0.19 (95% CI: −0.25 to 0.63), suggesting no significant long-term benefit or evidence of relapse. Heterogeneity was moderate (I^2^ = 79.1%) The forest plot for PAR score is shown in Figure 6.

#### 3.4.4. Certainty of Evidence (GRADE)

The overall certainty of evidence was assessed using the GRADE framework and varied across outcomes. The certainty was rated as moderate for both overjet and ANB angle, primarily due to downgrades for risk of bias (notably concerns related to outcome measurement and selective reporting), as well as inconsistency arising from heterogeneity in treatment protocols and follow-up durations. The certainty of evidence for the PAR score was rated as low, with downgrades attributed to serious imprecision, reflecting wide confidence intervals and small sample sizes in contributing studies, as well as methodological concerns related to study design and reporting. The complete summary of findings is presented in Table 1.

## 4. Discussion

This systematic review and meta-analysis evaluated the long-term stability and relapse associated with early orthodontic interventions initiated during the mixed dentition stage and provides a contemporary and comprehensive assessment of the evidence base. Although early treatment with fixed or removable appliances showed short-term improvements in dental and skeletal relationships, reflected by initial reductions in overjet, ANB angle, and PAR scores, these changes did not reliably translate into statistically significant or clinically meaningful long-term outcomes. The pooled estimates from meta-analyses of overjet (9 studies), ANB angle (6 studies), and PAR scores (4 studies) revealed wide confidence intervals and high heterogeneity, contributing to the lack of consistent findings or robust evidence of lasting benefits from early orthodontic intervention. These findings are consistent with previous research, which reported limited long-term skeletal benefits from early use of functional appliances [5,9,10]. Raucci et al. (2015) further emphasized that while interceptive orthodontics may offer short-term esthetic and psychosocial benefits, skeletal effects are often temporary and do not persist into adolescence [26].

### 4.1. Certainty of Evidence: Findings and Implications

Despite the application of the GRADE framework, the overall certainty of evidence regarding long-term stability was moderate for overjet and ANB angle, and low for PAR score, primarily due to imprecision, risk of bias, and heterogeneity. The inclusion of non-randomized studies, two of which were assessed as having an overall high risk of bias using the ROBINS-I tool, may have influenced the meta-analytic findings. In particular, bias due to confounding was a key concern, as these studies lacked adequate control for baseline skeletal or dental characteristics that could affect treatment outcomes. Additionally, selection bias and the absence of blinding in outcome assessment may have led to systematic differences in how outcomes were measured and reported. These factors may have contributed to an overestimation of treatment effects and increased heterogeneity, thereby reducing the internal validity of the pooled estimates. These limitations were explicitly considered in the GRADE evaluation, and the inclusion of these studies underscores the need for cautious interpretation of results, especially in the absence of high-quality randomized evidence for certain outcomes.

Furthermore, the very high I^2^ values observed likely reflect clinical heterogeneity (e.g., differences in appliance types and treatment protocols) and methodological variability across studies. This highlights the need for conducting adequately powered, standardized trials to enable more robust, appliance-specific meta-analyses and to enhance the generalizability of future findings. These GRADE assessments highlight the need for cautious interpretation of the pooled estimates, particularly in terms of long-term occlusal stability. The overall strength of evidence supports a possible benefit of early orthodontic intervention in improving certain skeletal and dental parameters, but variability in study quality and consistency limits the confidence in these conclusions. Future research with standardized outcome measures, longer follow-up, and improved methodological rigor is needed to strengthen the evidence base.

### 4.2. Long-Term Stability and Retention Strategies

The lack of significant long-term differences in PAR scores, ANB angle, and overjet aligns with earlier studies conducted by O’Brien et al. (2009) and Anne Mandall et al. (2012) which found that early intervention does not provide superior long-term stability in correcting Class II malocclusions [6,17]. While some studies reported modest retention of overjet correction [8,14], others such as Quinzi et al. and Baccetti et al. observed measurable relapse, particularly in skeletal parameters [12,18]. A key limitation in achieving long-term stability appears to be the variability in post-treatment retention strategies. Notably, fewer than half of the included studies explicitly reported retention protocols, limiting our ability to evaluate their impact on post-treatment stability [8,17,18]. Moreover, the lack of standardized outcome reporting and consistent follow-up protocols continues to limit the ability to make direct comparisons across studies. Citation network analysis further revealed a fragmented evidence base, with limited continuity between foundational studies and more recent trials, highlighting the need for a more cohesive and cumulative approach to research in this field.

The long-term stability of outcomes is influenced by both the malocclusion type and patient characteristics. Functional appliances play an important role in the early management of skeletal Class II malocclusions. When used during active growth, they can stimulate mandibular advancement, improve sagittal discrepancies, and enhance facial profile, thereby reducing the need for more complex treatment at later stages. However, their effectiveness depends on proper treatment timing and patient compliance, with reduced outcomes if growth potential is limited or adherence is poor [27]. In addition, craniofacial growth patterns and malocclusion prevalence vary across racial and ethnic groups. Recognizing such differences is important for diagnosis and treatment planning to ensure individualized and culturally appropriate care. Moreover, the risk of relapse after early treatment varies with etiology. Malocclusions with a strong genetic basis, such as severe Class II or Class III patterns, are more likely to relapse because they follow inherited growth patterns [28]. In contrast, environmentally influenced problems, such as those related to habits or airway obstruction, may remain stable once the underlying cause is removed [29].

### 4.3. Clinical Relevance

This review offers significant implications for clinical decision-making. Moderate-certainty evidence for the ANB angle suggests that early orthodontic intervention may contribute to favorable skeletal changes. In clinical practice, these findings support the use of early treatment in selected Class II cases to influence jaw growth. Nevertheless, treatment decisions should be individualized, given the variability in appliance types and patient growth patterns. Similarly, overjet reduction, also supported by moderate-certainty evidence, indicates that early intervention is effective in achieving dental improvements that may help reduce the risk of dental trauma and address psychosocial concerns. However, the long-term stability of these improvements remains uncertain due to the potential for relapse, underscoring the need for continued monitoring. In contrast, the evidence for PAR score, reflecting overall occlusal improvement, was rated as low certainty. This limits the strength of conclusions regarding long-term functional outcomes. While early treatment may provide short-term occlusal benefits, the current evidence does not reliably support its effectiveness in reducing the need for future treatment or achieving a stable long-term occlusion.

From a clinical perspective, the findings of this review suggest that early orthodontic treatment should not be routinely advised with the expectation of achieving long-term skeletal correction. However, the small, statistically non-significant mean differences observed (e.g., −0.18 mm in overjet) warrant cautious interpretation, as they may still hold clinical relevance in specific contexts. Early intervention may be appropriate in selected cases, such as those involving functional crossbite, risk of incisor trauma, or significant psychosocial concerns, where even minor occlusal improvements can offer meaningful benefits in terms of function, esthetics, or trauma prevention. The modest long-term impact observed on outcomes such as the ANB angle and PAR scores further underscores the importance of individualized, case-by-case decision-making. Given the increased duration, cost, and complexity associated with two-phase treatment protocols, early treatment should be considered only in cases with clearly defined clinical indications. Overall, these findings support a cautious, individualized approach to early orthodontic intervention. Clinicians should weigh potential skeletal and dental benefits alongside patient growth, compliance, and risk of relapse. The results underscore the importance of shared decision-making with families and long-term monitoring beyond the early treatment phase.

### 4.4. Quality of Evidence

The quality of the included studies varied, with randomized controlled trials generally demonstrating a low risk of bias, whereas non-randomized studies exhibited moderate risk due to confounding and selection bias. Although most RCTs were rated as low risk of bias, the inclusion of non-randomized studies in the meta-analyses may have introduced residual confounding and potentially diluted effect estimates, thereby limiting the robustness of the findings.

### 4.5. Strengths and Limitations

This systematic review adhered to PRISMA 2020 guidelines and incorporated several methodological strengths, including independent dual risk of bias assessments using RoB 2 and ROBINS-I, comprehensive search across major databases and gray literature sources, and the application of the GRADE framework to assess the certainty of evidence for all outcomes. Despite these strengths, study limitations should be noted. The included studies exhibited considerable clinical and methodological heterogeneity, lack of consistent definitions of outcomes and variable follow-up durations. The reliability of findings related to PAR scores was further limited by small sample sizes. Additionally, assessment of publication bias through funnel plot analysis was not feasible, as fewer than ten studies were available per outcome. Another limitation of this review was that initial title and abstract screening was conducted by a single reviewer, which may have introduced selection bias. However, this risk was mitigated by consulting an additional reviewer during the full-text screening stage for studies with unclear eligibility, with disagreements resolved through discussion and consensus. Thirdly, this review included only English-language publications, which may have introduced language bias by excluding relevant studies published in other languages. This limitation could potentially affect the evidence base and may reduce the global applicability of the findings.

### 4.6. Future Directions

Future research should aim to stratify participants by growth stage and severity of malocclusion, adopt standardized outcome criteria (such as defining clinically significant overjet relapse as ≥2 mm), and implement consistent retention protocols. Comparative studies should evaluate both clinical outcomes and patient-centered measures such as treatment acceptability, quality of life, and long-term cost-effectiveness. Additionally, the integration of advanced technologies, including three-dimensional imaging and AI-driven prediction models may further enhance treatment planning and forecasting post-treatment stability with greater precision.

## 5. Conclusions

Early orthodontic treatment during the mixed dentition phase provides short-term improvements in occlusal parameters such as overjet, ANB angle, and PAR scores. However, current evidence does not demonstrate a consistent long-term advantage compared to delayed or later intervention. Therefore, routine early treatment cannot be recommended solely for achieving long-term skeletal or dental stability. Clinical indications for early intervention should be highly selective, focusing on cases where timely correction reduces specific risks such as dental trauma, psychosocial concerns, or functional crossbite. Future research should prioritize methodological rigor, standardized outcomes, longer follow-up periods, and incorporation of patient-centered measures to strengthen the evidence base and inform individualized treatment planning.

## Figures and Tables

**Table 1:** GRADE Summary of Evidence for Overjet, ANB Angle, and PAR Score Outcomes.

Outcome	No. of Studies	Risk of Bias	Inconsistency	Indirectness	Imprecision	Publication Bias	Certainty
Overjet	8	Some concerns	Low	No	Some concerns	Undetected	Moderate
ANB Angle	4	Some concerns	Low	No	Some concerns	Undetected	Moderate
PAR Score	4	Some concerns	Low	No	Serious	Undetected	Low

## Data Availability

The original contributions presented in this study are included in the article.

## Supplementary Materials

The following supporting information can be downloaded at https://www.mdpi.com/article/10.3390/medicina61101854/s1: Table S1: Characteristics of Included Studies Evaluating Interceptive Orthodontic Treatment in Children; Table S2: Summary of Key Outcomes from Included Studies; Search strategy-Supplementary File.
