# Supplementary material for: Does Early Orthodontic Treatment in Mixed Dentition Improve Long-Term Outcomes? A Systematic Review and Meta-Analysis
Source: Medicina (Kaunas). 2025 Oct 16;61(10):1854. doi: 10.3390/medicina61101854 (PMC12565956; doi:10.3390/medicina61101854)
Supplement: Supplementary file 1 [file medicina-61-01854-s001.zip › Search strategy-Supplementary File.pdf]

Search Strategy (search string)

Pubmed

(orthodontic*[Title/Abstract] OR "interceptive orthodontics"[Title/Abstract] OR "early orthodontic treatment"[Title/Abstract] OR "phase I"[Title/Abstract])AND (stability*[Title/Abstract] OR relapse*[Title/Abstract] OR "long-term"[Title/Abstract] OR retention[Title/Abstract] OR overjet[Title/Abstract] OR ANB[Title/Abstract] OR "Peer Assessment Rating"[Title/Abstract] OR PAR[Title/Abstract]) AND(child*[Title/Abstract] OR pediatric*[Title/Abstract] OR paediatric*[Title/Abstract] OR "mixed dentition"[Title/Abstract])

Scopus

TITLE-ABS-KEY ((orthodontic* OR "interceptive orthodontics" OR "early orthodontic treatment" OR "phase I") AND (stability* OR relapse* OR "long-term" OR retention OR overjet OR ANB OR "Peer Assessment Rating" OR "PAR index" OR PAR) AND(child* OR pediatric* OR paediatric* OR "mixed dentition"))

Web of Science

TS=((orthodontic* OR "interceptive orthodontics" OR "early orthodontic treatment" OR "phase I") AND (stability* OR relapse* OR "long-term" OR retention OR overjet OR ANB OR "Peer Assessment Rating" OR "PAR index" OR PAR) AND (child* OR pediatric* OR paediatric* OR "mixed dentition"))

Google Scholar (screen first 100 results)

orthodontic OR "interceptive orthodontics" "mixed dentition" (stability OR relapse OR "long-term" OR retention OR overjet OR ANB OR "Peer Assessment Rating" OR PAR)

Grey literature details

Ovid platform searches for conference abstracts and non-indexed items using simplified versions of the above strings (no additional limits).
Theses/dissertations: ProQuest Dissertations & Theses Global, EThOS, and OATD using the phrase queries:
("interceptive orthodontics" OR "early orthodontic treatment") AND "mixed dentition"
orthodontic AND (stability OR relapse) AND (child* OR pediatric* OR paediatric*)
