# Supplementary material for: Does Early Orthodontic Treatment in Mixed Dentition Improve Long-Term Outcomes? A Systematic Review and Meta-Analysis
Source: Medicina (Kaunas). 2025 Oct 16;61(10):1854. doi: 10.3390/medicina61101854 (PMC12565956; doi:10.3390/medicina61101854)
Supplement: Supplementary file 1 [file medicina-61-01854-s001.zip › Table S1-Supplementary File.pdf]

Table S1: Characteristics of Included Studies Evaluating Interceptive Orthodontic Treatment in Children

S.no	Study, year, Country	Design	Population	Intervention	Comparator	Follow-up Duration	Outcome Measures	Key Findings
1.	Quinzi et al. (2023), Italy [12]	Retrospective cohort study	78 subjects; (mean age 8.5 ± 1.4 years); 52 treated subjects and 26 controls matched for age and observation time	Serial Extraction (EX) or Expansion + Extraction (EXP-EX)	Untreated control group	Until eruption of all permanent posterior teeth (mean not clearly specified but implied longitudinal)	Skeletal and dental changes on lateral cephalograms: mandibular inclination, occlusal plane angle, superior gonial angle, facial height index, incisor inclination	Both EX and EXP-EX led to vertical skeletal changes (reduced mandibular inclination, occlusal plane angle, superior gonial angle). No significant differences in sagittal skeletal parameters. Dental parameters less affected. Results emphasize vertical skeletal effects of both interventions.
2.	Dias et al. (2021), Brazil [13]	RCT	99 subjects; Children aged 7 to 10 years	bonded lingual spurs (BS), chin cup, fixed palatal crib (FPC)	Removable palatal crib (RPC).	2 years	Stability of early anterior open bite (AOB) treatment using different appliances in children	All 4 appliances are effective in treating anterior open bite, with stable results over a 2-year follow-up. The fixed palatal crib demonstrated the highest AOB correction and the lowest patient withdrawal rate, and breaking deleterious oral habits
3.	Keski-Nisula et al. (2020), Finland [14]	Controlled Trial	313 children; Aged 5–8 years (from Jalasjärvi and Kurikka); 255 treated with EGA,	Eruption Guidance Appliance (EGA)	Untreated controls	>10 years (mean age at follow-up was 16.7 years)	Overjet, overbite, molar relation, mandibular length, incisor crowding,	Significant and stable improvements in overjet, overbite, and molar relationships. Class II prevalence reduced from 100% to 14%. Mandibular

Table S1: Characteristics of Included Studies Evaluating Interceptive Orthodontic Treatment in Children

			58 untreated controls				cephalometric parameters	length increased more in treated group. Overbite increased by 0.9 mm; late lower incisor crowding in 14%. No second treatment phase required. Results remained stable into early permanent dentition. No cost-benefit analysis; sex imbalance and retention not fully addressed.
4.	Myrlund et al. (2014),Norway, Finland [15]	RCT	48 children aged 7–8 years; 25 in the EGA group and 23 in the control group; randomly selected from a pool of 159 screened patients	Eruption Guidance Appliance (EGA) during early mixed dentition	Untreated controls	1 year	Overjet, overbite, Angle's classification, anterior crowding	Significant improvements in overjet, overbite, molar relation, and anterior crowding in the treatment group. Deep bite reduced. No long-term follow-up data. Study had high measurement reliability (ICC = 0.95). Early treatment with EGA was effective but longer duration may be needed for full correction. No differences at baseline. Follow-up data needed for assessing long-term stability.
5.	Lippold et al., 2013, Germany [16]	RCT	66 children with unilateral posterior crossbite (31 treatment	Bonded maxillary expansion device followed by U-	Untreated control group	1 year	Intercanine distance, transverse widths, basal arch length, midline deviation,	Early orthodontic treatment significantly improved intercanine and transverse maxillary

Table S1: Characteristics of Included Studies Evaluating Interceptive Orthodontic Treatment in Children

			group, 35 control group); mean age 7.3 ± 2.1 years	bow activator therapy			vertical overbite, craniofacial growth patterns	widths, basal arch length, and vertical overbite. Midline deviation corrected in the therapy group. Post-treatment craniofacial growth prognosis was enhanced. Results support early treatment with bonded expansion followed by U-bow activator therapy. Randomization applied; however, dropout rates and lack of full blinding may affect generalizability.
6.	Anne Mandall N et al. (2012),UK [17]	RCT	73 children aged below 10 years (PFG: n=35, CG: n=38; stratified randomization by gender; final analyzed sample: 46)	Early Class III protraction facemask therapy	Untreated controls	3 years	ANB angle, overjet, PAR score, TMJ signs, self-esteem (Piers-Harris scale), psychosocial impact (OASIS questionnaire)	Early protraction facemask treatment improved ANB by 1.5° and overjet by 3.6 mm; 70% of PFG patients maintained a positive overjet. The treatment group showed a 21% PAR score improvement versus an 8.4% worsening in controls. No significant differences were found in self-esteem or psychosocial impact. No TMJ-related side effects observed. Limited blinding and dropout bias

Table S1: Characteristics of Included Studies Evaluating Interceptive Orthodontic Treatment in Children

								noted; intention-to-treat analysis used to mitigate this.
7.	King et al. (2012), USA [8]	RCT	134 Medicaid dental and medical patients with mixed dentition; IO group (n=65), CO group (n=69); mean dropout 21%. Children 8–11 years	Interceptive orthodontics	Comprehensive Treatment later	4 years	Peer Assessment Rating (PAR), Index of Complexity, Outcome and Need (ICON), treatment need, dropout, generalizability	IO was effective in reducing malocclusions in Medicaid patients, but less so than CO. At 48 months, CO achieved greater PAR reduction (18.6 points vs 10.1 in IO). Both groups saw significant reduction in treatment need. IO offers a less complex and lower-cost alternative for low-income populations but may be less effective for complex cases. Dropout rate was 21%, comparable to other RCTs. Patient selection is critical for IO effectiveness. Trial was limited by socioeconomic generalizability and unfinished cases.
8.	Baccetti et al. (2011), Italy [18]	RCT	120 subjects in late mixed dentition with palatally displaced canines (PDCs); enrolled	Transpalatal arch (TPA) + extraction of deciduous canine ± rapid maxillary expansion (RME)	Untreated controls	2 years	Successful eruption of PDCs, confirmed by radiographs and cephalograms; assessed based on treatment modality	Successful eruption observed in 80% (RME/TPA/EC) and 79.2% (TPA/EC). Prevalence significantly higher in treated groups than controls.

Table S1: Characteristics of Included Studies Evaluating Interceptive Orthodontic Treatment in Children

			at the University of Florence					Unsuccessful cases showed advanced root development. Interceptive treatment more effective before root apex closure. Radiographic predictors like CVM stages and root apex development were useful prognostic tools.
9.	Jolley et al. (2010), USA [19]	RCT	170 Medicaid-enrolled children referred from community clinics; randomized using block allocation	Interceptive orthodontics	Untreated observation	2 years	Peer Assessment Rating (PAR), Medicaid eligibility status, percentage achieving 70% PAR reduction	81% of interceptive patients no longer met the "medically necessary" Medicaid treatment criteria. Only 24% achieved a 70% PAR reduction, suggesting partial effectiveness. Interceptive treatment may reduce severity of malocclusion and improve access in underserved populations but may not eliminate need for comprehensive treatment. Long-term cost-effectiveness and eligibility impact require further evaluation.

Table S1: Characteristics of Included Studies Evaluating Interceptive Orthodontic Treatment in Children

10.	O'Brien et al. (2009), UK [6]	RCT	120 children with Class II Division 1 malocclusion; 60 in early treatment group, 60 in delayed treatment group; minimum overjet of 7 mm. Children aged 8-10 years	Early treatment using Twin-block appliance	Delayed treatment (starting at 12.4 years)	10 years	Peer Assessment Rating (PAR), cost, treatment duration, patient satisfaction, self-esteem, skeletal pattern	Early treatment resulted in higher PAR scores (worse outcome), longer treatment time, and ~$900 higher cost. No significant differences in skeletal changes or self-esteem. 15% of early-treatment patients avoided later complex treatment. Overall, early functional treatment with Twin-block showed no significant long-term advantage over adolescent-only treatment. Informed decision-making and cost-benefit considerations are essential.
11.	Keski-Nisula et al. (2008), Finland [20]	Prospective Controlled Cohort Study	255 children (treatment group) from Jalasjärvi and Kurikka; 104 untreated controls from Seinäjoki; Children in early mixed dentition stage (age)	Eruption Guidance Appliance (EGA)	Untreated controls	3.3 years	Overjet, overbite, crowding, Class I relationship, occlusal deviations	EGA significantly reduced overjet and overbite; 99% of treated children achieved tooth contact vs 24% in controls. Class I canine/molar achieved in 90% of treated vs 48% controls. Only 13% of treated had occlusal deviations compared to 88% controls. Compliance

Table S1: Characteristics of Included Studies Evaluating Interceptive Orthodontic Treatment in Children

								issues noted (31% discontinued due to cooperation). Early intervention effective in reducing treatment need, particularly with strong family support.
12.	Krušinskienė et al. (2008), Lithuania, Finland [21]	Prospective Randomized Controlled Trial	68 children (40 males, 28 females), aged 7.6 ± 0.3 years; Class II tendency and moderate crowding	Early Headgear (HG) treatment	Control group (delayed/no treatment)	13 years	Peer Assessment Rating (PAR), Little's Irregularity Index (LII), intercanine distance, occlusal stability	No significant difference in long-term occlusal outcomes between HG and control groups at 13-year follow-up. Patients treated without extractions had better PAR scores and alignment. Extraction-based treatments showed more lower incisor irregularity. Treatment timing had minor influence on long-term aesthetics. Study emphasized limitations in predicting post-retention relapse and recommended continued monitoring and use of objective indices like PAR.

Table S1: Characteristics of Included Studies Evaluating Interceptive Orthodontic Treatment in Children

13.	*Torres et al. (2006), Brazil [22]	Prospective Controlled Trial	60 children (aged 6–10 years) with Angle Class I anterior open bite (AOB); 30 treatment group and 30 untreated control group.	Removable appliance with palatal crib + high-pull chin cup	Untreated controls	1 year	Overbite, molar eruption, dentoalveolar and soft tissue changes (via cephalometric analysis)	Treated group showed significant dentoalveolar changes and mean AOB closure of 3.86 mm. No significant changes in molar eruption or lower anterior face height. Vertical control by chin cup was limited. Significant increase in overbite observed, attributed to dental rather than skeletal effects. The study emphasized the role of incisor movement and need for longer follow-up to assess stability. Skeletal changes were minimal.*
14.	Kau et al. (2004), Italy, Germany, Wales [23]	RCT	83 children (aged 6–10 years) from dental clinics in South Wales, Italy, and Germany; randomized to extraction or non-extraction.	Primary canine extraction	Observation	1–2 years	Lower incisor crowding, arch length, molar migration	Extraction group showed 4.76 mm greater reduction in lower incisor crowding than controls but also 2.73 mm greater arch length loss, indicating forward molar migration. Only ~25% of extraction cases showed crowding improvement; crowding improvement odds estimated at 1 in 4. Limited predictability of success; findings raise

Table S1: Characteristics of Included Studies Evaluating Interceptive Orthodontic Treatment in Children

								concern about the routine use of primary canine extraction for crowding relief.
15.	Mäntysaari et al. (2004), Finland [24]	RCT	Children aged 7.6 years; Screened for moderate crowding and Class II tendency; randomized to 2 groups	Cervical headgear for 16 months	Untreated controls	2 years	Arch length/width, incisor inclination, SNA angle, anterior facial height (cephalogram + model casts)	Headgear group showed significant increases in arch width and length, and labial inclination of incisors. Maxillary growth was restrained (SNA angle decreased). Overjet not corrected by headgear alone. Control group received interceptive treatment (primary canine extractions in ~36% cases, stripping in ~19%). Early cervical headgear is effective for moderate crowding management, but overjet correction needs adjunctive treatments.
16.	Tulloch et al. (2004), USA [5]	Multiphase RCT (Parallel Design)	166 children with Class II malocclusion, overjet ≥7 mm, no prior orthodontic treatment; randomized into	Early 2-phase treatment (phase 1: early ortho + later fixed appliance)	Single-phase treatment (fixed appliance in adolescence)	5–7 years	Overjet, skeletal/dental measures, cephalometrics, extraction frequency, treatment duration	Early treatment led to favorable growth changes in 75% but had no long-term advantage in treatment outcome. No significant differences in skeletal/dental results or

Table S1: Characteristics of Included Studies Evaluating Interceptive Orthodontic Treatment in Children

			early or late treatment arms; Children aged ~9 years					fixed appliance duration at end of phase 2. Efficiency of 2-phase treatment is not superior. Early treatment should be reserved for specific indications rather than general use. Results emphasize caution against relying on clinical impressions alone.
17.	O'Brien et al., 2003, UK [25]	RCT	174 children aged 8–10 years with Class II Div 1 malocclusion; overjet ≥7mm; NHS-based specialists	Twin-block functional appliance (modified design)	Untreated control group	15 months	Overjet, PAR scores, cephalometric skeletal and dental measures	Early Twin-block treatment significantly reduced overjet and PAR scores (~42% reduction). Most improvement was dentoalveolar; skeletal change minimal. Initial discrepancy magnitude predicted treatment effect. 16% of treatment group didn't complete therapy. Results are applicable to real-world NHS settings. Supports early functional appliance use for overjet reduction but not for major skeletal correction.

Table S1: Characteristics of Included Studies Evaluating Interceptive Orthodontic Treatment in Children

18.	Tulloch et al. (1998),USA [10]	RCT	166 children with Class II malocclusion (147 completed phase 2); preadolescent; randomized by blocks	Early treatment with headgear or functional appliance, followed by comprehensive fixed appliance	One-stage later comprehensive treatment	5–7 years	ANB angle (skeletal), PAR scores (occlusion), total treatment duration	Early treatment reduced skeletal discrepancies in ~75%, but skeletal effects not maintained after full treatment. Total treatment time longer with two-phase approach. No significant difference in final occlusion or skeletal relationships between early and later treatment. Compliance and clinician skill influenced outcomes. PAR improvements modest; variability in outcomes suggests individualized planning is needed.

*Note: Torres et al. (2006) [22] primarily reported dentoalveolar open bite correction rather than skeletal stability, but met minimum inclusion criteria for interceptive treatment and follow-up.
