# Supplementary material for: Does Early Orthodontic Treatment in Mixed Dentition Improve Long-Term Outcomes? A Systematic Review and Meta-Analysis
Source: Medicina (Kaunas). 2025 Oct 16;61(10):1854. doi: 10.3390/medicina61101854 (PMC12565956; doi:10.3390/medicina61101854)
Supplement: Supplementary file 1 [file medicina-61-01854-s001.zip › Table S2-Supplementary File.pdf]

Table S2. Summary of Key Outcomes from Included Studies

S.no	Study, Year, Country	Design	Outcome Measures
1.	Quinzi et al. (2023), Italy [12]	Controlled Retrospective cohort study	• The study compared skeletal and dental effects of severe crowding treatment by serial extractions or maxillary expansion in mixed dentition<br>• Both treatment modalities significantly affected vertical skeletal parameters, decreasing mandibular inclination and occlusal plane inclination<br>• A significant decrease in the superior part of the gonial angle was observed in both extraction groups<br>• No significant differences were found in sagittal skeletal parameters between the groups at baseline<br>• The annualized changes in the superior gonial angle significantly differed among the Control, EX, and EXP-EX groups<br>• Dental parameters were considerably affected by treatment compared to untreated controls
2.	Dias et al. (2021), Brazil [13]	RCT	• Multi-arm RCT in early/mixed dentition compared four approaches for anterior open bite (AOB): bonded spurs (BS), chin cup (CC), fixed palatal crib (FPC), and removable palatal crib (RPC).<br>• All appliances corrected AOB at end of treatment (T2); stability differed by appliance at 2-year follow-up (T3).<br>• At T3, mean overbite (mm) indicated best stability with FPC (≈ +1.23 mm), followed by RPC (≈ +0.73 mm); BS showed minimal positive overbite (≈ +0.19 mm), while CC showed slight relapse to open bite (≈ −0.19 mm).<br>• Retention to T3 was 63/99 analyzed (BS 15; CC 11; FPC 21; RPC 16); mean age at T3 ≈ 11.4–11.7 years.<br>• No untreated control arm; between-group differences at T3 favored crib-based appliances (FPC/RPC) for overbite stability.<br>• Heterogeneity in protocols/retention reporting limited appliance-specific secondary outcomes; overjet and skeletal (ANB) changes were not consistently sustained relative to dental overbite correction.
3.	Keski-Nisula et al. (2020), Finland [14]	Controlled Trial	• Class II treatment with an EGA significantly improved molar relationships, overjet, and overbite in early mixed dentition<br>• The treatment group showed a decrease in Class II frequency from 100% to 14% during treatment<br>• Overbite increased by 0.9 mm, while late crowding of lower incisors was observed in 14% of children<br>• Mandibular length increased significantly more in the treatment group compared to controls<br>• No children required a second treatment phase after early intervention<br>• The results remained stable into early permanent dentition at age 16.7 years
4.	Myrlund et al. (2014),Norway, Finland [15]	RCT	• The mean ICC for duplicate cephalometric measurements was 0.95, indicating high reliability<br>• Significant decreases in overjet and overbite were observed in the treatment group

Table S2. Summary of Key Outcomes from Included Studies

			• Class II molar relationships decreased significantly in the treatment group<br>• The number of children with impinging deep bite significantly reduced in the treatment group<br>• No significant differences were found between treatment and control groups at T1<br>• Follow-up data are needed to assess long-term effects of the treatment<br>• The study involved 48 children, with 46 completing the study.
5.	Lippold et al., 2013, Germany [16]	RCT	• The therapy group showed significant maxillary expansion in intercanine distance and transversal widths between T1 and T2<br>• The control group exhibited significant growth in intercanine distance and anterior transversal width<br>• A very significant difference was noted between therapy and control groups for maxillary growth at T2<br>• The therapy group experienced a significant increase in basal arch length across all regions.<br>• Midline deviation significantly improved in the therapy group, while it slightly increased in the control group<br>• Vertical overbite increased significantly in the therapy group, contrasting with stability in the control group<br>• Overall, orthodontic treatment improved dental occlusion and enhanced craniofacial growth prognosis
6.	Anne Mandall N et al. (2012),UK [17]	RCT	• The study found that early class III protraction facemask treatment resulted in significant improvements in ANB (1.5°) and overjet (3.6 mm) compared to the control group, which showed minimal changes.<br>• The treatment group demonstrated a 21% improvement in the peer assessment rating (PAR), while the control group worsened by 8.4%.<br>• However, there was no statistically significant increase in self-esteem or reduction in the psychosocial impact of malocclusion between the groups at the 3-year follow-up.<br>• Overall, 70% of patients in the treatment group maintained a positive overjet, indicating ongoing treatment success.
7.	King et al. (2012), USA [8]	RCT	• The study found that initial orthodontic (IO) is effective at reducing malocclusions in Medicaid patients, but less so than Comprehensive orthodontic (CO)<br>• At 48 months, CO showed significant improvements in PAR and ICON compared to IO.<br>• IO had a mean reduction of 10.1 PAR points, while CO had 18.6 PAR points.<br>• Both groups experienced significant reductions in need by 48 months, with CO showing less need than IO.<br>• The dropout rate was 21%, which was less than expected.<br>• Baseline characteristics of dropouts did not significantly differ from completers.

Table S2. Summary of Key Outcomes from Included Studies

8.	Baccetti et al. (2010), Italy [18]	RCT	• The study found a successful eruption rate of 80% for the RME/TPA/EC group and 79.2% for the TPA/EC group<br>• A total of 40 subjects were in the RME/TPA/EC group, with 66 PDCs<br>• The prevalence of successful eruption was significantly higher in treated groups compared to the control group<br>• Dropouts did not affect the study's power, with three subjects relocating during the observation period<br>• The success rate for RME/TPA or TPA treatment was 75%, similar to other methods<br>• Unsuccessful cases showed more advanced root development compared to successful cases<br>• The study concluded that TPA with deciduous canine extraction is an effective treatment for palatally displaced canines
9.	Jolley et al. (2010), USA [19]	RCT	• The interim report compares dental outcomes by evaluating PAR scores after interceptive treatment or observation<br>• 81% of interceptive patients were no longer considered "medically necessary" for further treatment<br>• Only 24% of interceptive patients achieved a 70% reduction in PAR score, indicating significant improvement<br>• The study highlights the need for cost-effectiveness analysis between interceptive and comprehensive treatments<br>• Results suggest that further analysis is required as the trial progresses and more data becomes available
10.	O'Brien et al. (2009), UK [6]	RCT	• Early treatment resulted in higher PAR scores at the end of treatment compared to later treatment ($P = 0.002$)<br>• Patients receiving early treatment had significantly longer overall treatment times than those treated later ($P < 0.001$)<br>• The cost of early treatment was approximately $900 more than later treatment ($P < 0.001$)<br>• There were no significant differences in skeletal pattern, extraction rates, or self-esteem between early and later treatment groups<br>• Early treatment increased patient attendances and duration of treatment, leading to poorer final occlusion<br>• The study concluded that early treatment offers minimal benefits over treatment starting in adolescence
11.	Keski-Nisula et al. (2008), Finland [20]	Prospective Controlled Cohort Study	• Eruption guidance appliance (EGA) treatment significantly reduced overjet and overbite.<br>• 99% of treated children achieved tooth contact compared to 24% in the control group.<br>• Class I canine and molar relationships were achieved in 90% of treated children versus 48% in controls.<br>• Only 13% of treated children had occlusal deviations, compared to 88% in the control group.<br>• Compliance issues were noted, with 31% of children discontinuing due to cooperation challenges.<br>• Early intervention with EGA was effective in reducing long-term treatment need, especially when there was strong family support.

Table S2. Summary of Key Outcomes from Included Studies

12.	Krušinskienė et al. (2008), Lithuania, Finland [21]	Prospective Randomized Controlled Trial	• The study involved 68 children, with no significant differences in occlusal outcomes between HG treatment and control groups at 13 years follow-up<br>• Lower PAR scores were noted in patients treated without extractions, indicating better outcomes in this group<br>• The greatest irregularity in lower incisor alignment was found in subjects treated with extractions before treatment .<br>• The study concluded that treatment timing has a minor influence on stability, with no significant differences in long-term aesthetics<br>• The Peer Assessment Rating (PAR) Index effectively evaluated occlusal stability throughout the study
13.	Torres et al. (2006), Brazil [22]	RCT (Prospective Controlled Trial)	• The study found no intra-group sexual dimorphism between genders in either group<br>• Only nine out of 34 analyzed variables showed statistically significant changes<br>• The treated group had a mean AOB closure of 3.86 mm<br>• The control group exhibited less extrusion and tipping of incisors compared to the treated group<br>• No significant differences were observed in molar eruption or mesial movement<br>• Dentoalveolar changes were significant, but soft tissue changes were not<br>• The treatment resulted in an increase of overbite in all treated individuals
14.	Kau et al. (2004), Italy, Germany, Wales [23]	RCT	• Lower incisor crowding reduced by 4.76 mm more in the extraction group than in controls.<br>• Arch length decreased by 2.73 mm in the extraction group, suggesting forward molar migration.<br>• Only 1 in 20 extraction cases showed meaningful crowding improvement, questioning routine use.<br>• Net space gain from primary canine extraction was 2.03 mm.
15.	Mäntysaari et al. (2004), Finland [24]	RCT	• Early headgear (HG) treatment significantly affects dental arches and craniofacial morphology in children with moderate crowding .<br>• In the HG group, the mean treatment time was 8 months at T1 and 16.2 months at T2<br>• The control group underwent interceptive procedures, including extractions of primary canines in 38% and 35% of subjects, respectively<br>• Interdental stripping was performed in 19% of patients in the control group<br>• The results indicate a decrease in the SNA angle and increased anterior face height
16.	Tulloch et al. (2004), USA [5]	RCT	• Early treatment showed favorable growth changes in about 75% of children, but had little effect on subsequent treatment outcomes<br>• By the end of phase 2, no significant differences were found among treatment groups for skeletal and dental measures<br>• The average treatment time in fixed appliances was similar across groups, indicating no efficiency gain from early treatment<br>• The percentage of children requiring extractions approached significance, but was not statistically different among groups

Table S2. Summary of Key Outcomes from Included Studies

			• Cooperation was not the major factor influencing treatment outcomes, suggesting other variables played a role<br>• The study concludes that early treatment should not be considered efficient for most Class II children
17.	O'Brien et al., 2003, UK [25]	RCT	• The study involved 174 children with Class II Division 1 malocclusion, divided into treatment and control groups<br>• Twin-block treatment significantly reduced overjet and severity of malocclusion<br>• The treatment group showed a mean percentage reduction of 42% in PAR scores<br>• Most corrections were due to dentoalveolar changes, with minor skeletal changes.<br>• One patient in the control group was lost to follow-up, while 16% in the treatment group did not complete treatment<br>• The initial discrepancy magnitude influenced treatment outcomes<br>• The last data collection occurred in November 2000, 15 months post-treatment
18.	Tulloch et al. (1998),USA [10]	RCT	• Early treatment with headgear or functional appliances reduced skeletal discrepancies in children with Class II problems<br>• 75% of treated children showed favorable skeletal responses, while 25% did not<br>• No significant differences in jaw relationships or dental occlusion were found between early and later treatments<br>• Treatment time in fixed appliances was shorter for early treatment, but total treatment time was longer when including phase 1<br>• Variability in treatment response was influenced by factors like compliance and clinician proficiency<br>• The PAR scoring system indicated modest improvements in occlusion, with some patients experiencing worse outcomes<br>• The skeletal effects of early treatment were not maintained after comprehensive treatment
